# Molecular Characterization of Whole-Genome SARS-CoV-2 from the First Suspected Cases of the XE Variant in the Lazio Region, Italy

**DOI:** 10.3390/diagnostics12092219

**Published:** 2022-09-14

**Authors:** Martina Rueca, Emanuela Giombini, Giulia Gramigna, Cesare Ernesto Maria Gruber, Lavinia Fabeni, Angela Corpolongo, Valentina Mazzotta, Luisella Corso, Ornella Butera, Maria Beatrice Valli, Fabrizio Carletti, Stefano Pignalosa, Francesco Vairo, Emanuele Nicastri, Andrea Antinori, Enrico Girardi, Francesco Vaia, Fabrizio Maggi, SARS CoV-2 Lazio Surveillance Study Group

**Affiliations:** 1 National Institute for Infectious Diseases “Lazzaro Spallanzani” (IRCCS), 00149 Rome, Italy; 2 Department Clinical Pathology, S.M. Goretti Hospital, 04100 Latina, Italy

**Keywords:** COVID-19, SARS-CoV-2, whole-genome sequencing, recombination, PANGOLIN, Nextclade, XE

## Abstract

We report two cases of SARS-CoV-2 recombinant variant XE detected in nasopharyngeal swabs (NPS) of hospitalized patients with no evident epidemiological link in Lazio, Central Italy. Whole-Genome Sequencing (WGS) performed on an Ion Torrent GSS5 platform according to Italian flash surveys showed genomes corresponding to the PANGOLIN unclassified lineage and the Nextclade XE clade. Further analyses were then carried out to investigate more deeply the genetic characteristics of these XE-like sequences. When phylogenetic trees, by using IQ-TREE, were built splitting the genome into two regions according to the putative XE recombination site, the upstream and downstream regions were seen to be clustered near BA.1 and BA.2 sequences, respectively. However, our XE-like sequences clustered separately, with a significant bootstrap, from the classified European and Italian XE strains, although the recombination site between BA.1 and BA.2 was identified at the nucleotide site 11556 by RDP4 software, consistent with the putative XE breakpoint. These findings show the risk of the introduction of novel recombinant variants of SARS-CoV-2 and the existence of XE-like strains, phylogenetically separated, that could make their exact taxonomy difficult. It follows the need for continued SARS-CoV-2 surveillance by WGS.

## 1. Introduction

RNA viruses exhibit an extraordinary ability to produce genomic mutations, thus extending their genetic variability and acquiring adaptive advantages in response to host immunity and external pressures such as vaccination and antiviral drug therapies. Recombination is one of the most important mechanisms contributing to the genetic evolution of the viral genomes; it can occur both in non-segmented and segmented RNA viruses, when two or more viruses and/or strains of the same virus co-infect a single host cell [1,2]. Probably because of the way they replicate their genomes, recombination is described to occur at a higher frequency in positive-sense RNA viruses, a group that includes SARS-CoV-2 (Severe Acute Respiratory Syndrome Coronavirus, 2) and other clinically important seasonal coronaviruses [3,4]. Among these latter coronaviruses (e.g., HKU1, NL63, OC43, 229E), as well as MERS-CoV (Middle East Respiratory Syndrome Coronavirus), recombination events are common and, interestingly, occur mainly in the non-ORF1 region of the viral genome that encodes for structural genes [5,6]. As such, recombination can be considered an almost normal step of coronaviruses replication being required to produce the sub-genomic mRNAs and associated with changes in virulence and transmission of more-fit variants.

### 1.1. SARS-CoV-2 Recombination Events and Evolutionary Pathways

In the SARS-CoV-2 pandemic context, such an event may have major implications, particularly if new recombinants emerge with the property of escaping both natural and vaccine-induced immunity. Many studies have investigated the potential role of recombination in the zoonotic spillover of SARS-CoV-2, hypothesizing that the recombination event between different animal strains of beta-coronaviruses could be the crucial step by which the virus acquired the ability to infect humans [7,8]. On the contrary, studies focused on the characterization of recombinant strains’ emergence during the SARS-CoV-2 pandemic are still limited. Since the beginning of the COVID-19 (Coronavirus disease 2019) pandemic in 2019, SARS-CoV-2 revealed a genetically high variability with the emergence of multiple variants, of which several are considered variants of concern (VOC), given their impact on public health [9]. Each strain, from the Alpha to the Omicron variant, genetically differed from the previous one for insertion/deletion and point mutations that brought amino acid substitutions, principally in the spike protein-coding region of the viral genome [10,11,12,13]. More recently, several variants derived from the recombination process have been observed [14,15,16], and some of them are also described in Italy [17]. However, there are a limited number of publications reporting recombination events for circulating SARS-CoV-2, probably because recombination events can be difficult to detect whenever the SARS-CoV-2 sublineages are minimally different and require Whole-Genome Sequencing. Evidence exists on the importance of genomic epidemiology of SARS-CoV-2 as a public health tool; thus, failing to identify the recombinant strains could lead to an incorrect epidemiological inference on the real circulation of the SARS-CoV-2 variants.

### 1.2. Recombination Events Involving Omicron Variant

The first case of the recombinant variant involving the Omicron VOC was reported in France in January 2022; this variant was assigned as PANGO XD lineage (AY.4/BA.1) and classified by WHO as a variant under monitoring on 9 March 2022 [18]. During the same period, two other recombinant variants were described in the UK, such as XF (Delta/BA.1) and XE (BA.1/BA.2) [19]. Most Omicron recombinants identified to date have BA.1 as an acceptor with the breakpoint site in the ORF1ab region, and hence preserve the S region encoding Spike protein of BA.2 (e.g., variant XE, XG, XH, XJ, XK, XM, XN, XP, XQ, and XR). XP is the lone exception, having BA.1.1 as an acceptor (including Spike) and BA.2 as a donor. The highest spread of recombinant variant preserving BA.2-like Spike protein could be explained by an apparent increased transmissibility of BA.2 with respect to BA.1 lineage [20], together with the evidence of the BA.2 Spike protein structure being more stable than BA.1, making it less accessible to antibodies [21].

Among SARS-CoV-2 recombinants, XE is particularly interesting, having a growth rate advantage over BA.2 estimated from +9.8% [22] to about +21% [23]. The first case of XE was reported in the United Kingdom on 19 January 2022, rising to an incidence of about 763 daily cases on March 22. Soon after, XE started spreading to other countries all over the world such as Thailand, New Zealand, Israel, Cambodia, and India, with the first case reported on 8 April 2022. According to UKHSA and WHO agencies, more evidence is needed to study the variant’s transmissibility, eventually vaccine escape, and severity of symptoms [24]. The SARS-CoV-2 XE variant is a recombinant cross between two strains of the Omicron variant, namely BA.1 and BA.2. The XE variant contains the first portion of the genome of the Omicron BA.1 variant (up to 11,537 nucleotides), and the last major segment of the genome of the Omicron BA.2 variant (after the 11,537-nucleotide position), but contains NSP1-6 mutations derived from BA.1 and the rest of the genome derived from the BA.2 genome. At the time of writing, more than 2200 European XE sequences had been submitted to GISAID, with eight XE sequences found in Italy (six sequences in the Veneto region, one sequence in the Friuli Venezia Giulia region, and one sequence in the Piemonte region).

During the Italian genomic surveillance activity, between April and May 2022, we identified two SARS-CoV-2 strains belonging to the XE lineage. Interestingly, the two cases came from different areas of the Lazio region and had no evident epidemiological link. In this regard, we here describe the first XE cases identified in Central Italy and their genomic characteristics by Whole-Genome Sequencing and phylogenetic analysis.

## 2. Materials and Methods

Two nasopharyngeal swabs (NPS) were collected on 24th April and 25th May 2022, respectively, and analysed for SARS-CoV-2 detection in two different laboratories: at the Laboratory of Virology, INMI (Patient 1, Pt 1) and at “Santa Maria Goretti” Hospital in Latina (Rome) (Patient 2, Pt 2). The first NPS was collected from an 81-year-old female patient, not vaccinated, and affected by dementia (Pt 1). She was hospitalized for pneumonia at the National Institute for Infectious Diseases (INMI, Rome), and fully recovered 15 days after symptoms onset. The second case was a vaccinated 59-year-old male patient (Pt 2) who was admitted at “Santa Maria Goretti” Hospital in Latina (Rome) after a syncopal episode. He reported fever, myalgia, arthralgia, rash, asthenia, anorexia, and cough as early symptoms. The patient was discharged eight days after the onset of the symptoms. Table 1 summarizes the demographic and epidemiological characteristics of the patients.

The presence of SARS-CoV-2 RNA was detected by commercial RT-PCR assays: NPS collected from Pt 1 was analyzed by Alinity m SARS-CoV-2 Assay (Abbott, Chicago, Illinois, United States) targeting RdRp and N genes with a Ct value of 18.63; NPS collected from Pt 2 was analyzed by Allplex 2019-nCov Assay (Seegene, Seoul, South Korea) targeting E, RdRp and N genes (Ct values of 21.29, 22.44 and 21.40, respectively). Whole-Genome Sequencing was performed on available residual NPS samples. Nucleic acid extraction was performed by QiaSymphony automatic extractor (QIAGEN, Hilden, Germany) using a DSP Virus/Pathogen Kit (QIAGEN), starting from 600 uL and eluting in 70 uL of AVE buffer. Sequencing libraries were prepared by using Ion AmpliSeq SARS-CoV-2 Insight Research Assay, and Next-Generation Sequencing (NGS) was carried out on the Ion Torrent Gene Studio S5 Prime (GSS5 Prime) platform, following the manufacturer’s instructions (ThermoFisher, Waltham, MA, USA). Ethical approval for sequence analysis was obtained (No. 214/2020).

Mean quality Phred score >20 raw reads were selected and trimmed using Trimmomatic software v.0.36 [25]. SARS-CoV-2 genomes were assembled using the Easy-to-use SARS-CoV-2 Assembler pipeline (ESCA): a novel reference-based genome assembly pipeline specifically designed for SARS-CoV-2 data analysis [26], assembled genomes were also controlled using a second assembler software [27] and using Geneious Prime v.2019.2.3 for comparing assemblies.

For phylogenetic analysis, full-length SARS-CoV-2 sequences available on 13 June 2022 were retrieved from GISAID [28] and analyzed as follows. All Italian and European sequences with high coverage classified as XE PANGO lineage were considered in the analysis. Additionally, a selection of Italian complete sequences belonging to Omicron BA.1 and BA.2 variant lineages collected from April to March was also included. Moreover, other sequences with “unclassified” lineage but similar to the INMI XE were selected using the Audacy Instant tool on GISAID using default parameters (i.e., distance ≤3 with INMI sequences and ≥ 0.9 minimum quality distance score). Multi-sequence alignment was performed with MAFFT v7.271 [29], whole-genome alignment was manually controlled and 5′ and 3′ UTR regions were excluded from further analysis. Multiple phylogenetic analyses were performed to better investigate the features of the recombinant XE lineage. Three phylogenetic trees were built by using the whole-genome alignment and the upstream and downstream parts of the XE recombination site (nucleotide position 11,537). Maximum likelihood (ML) phylogenetic analysis was performed with IQ-TREE v.1.6.12 [30]; the best tree model was selected using ModelFinder [31]; the best trees were found performing 5000 bootstrap ultrafast replicates. Strain ILSGS00941 was used as a phylogenetic outgroup (PANGO lineage B.1.617.2, EPI_ISL_1663516).

Using the indication of the tree analysis, a set of BA.1, BA.2, and two representative sequences of BA.4 and BA.5 were used to verify the presence of recombination events in the XE INMI sequences. The recombination analysis was carried out by using the software Recombination Detection Program version 4 (RDP4) [32], including all sequences.

Moreover, the sequences were divided into groups (INMI XE, European XE, Italian XE, BA.1, and BA.2) to evaluate the genetic distance inter/intra group using MEGA-X (MEGA11: Molecular Evolutionary Genetics Analysis version 11) [33]. For each group, two distinct regions were tested as reported in the phylogenetic analysis (first part, up to the 11,537-nucleotide position, and the second part, after the 11,537-nucleotide position), in addition to the entire genome. All of the analysis was performed using the p method with 500 repetitions.

## 3. Results

During routine Whole-Genome Sequencing performed by the INMI Laboratory of Virology, according to Italian flash surveys, SARS-CoV-2-positive NPS samples from two hospitalized patients without any known epidemiological link were sequenced, obtaining on average about 250.000 reads per sample with a mean coverage of about 1700x. For each sample, the complete-genome sequences were confirmed using in parallel two different assembly software programs. Sequences were then deposited on GISAID with Accession IDs EPI_ISL_12739848 and EPI_ISL_13300253. The whole-genome sequences showed mutations corresponding to both Omicron BA.1 and BA.2 sublineages, resulting as “unclassified” after PANGOLIN analysis and classified into the XE clade by the Nextclade program.

**Figure 1:**
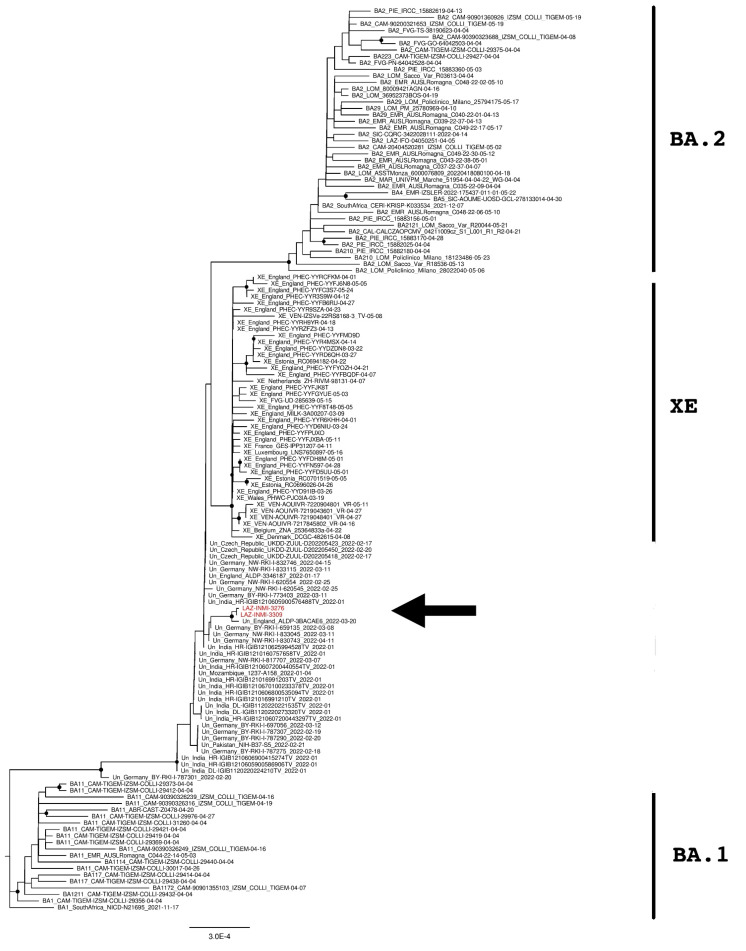
The phylogenetic tree is constructed by the maximum likelihood method and based on the whole-genome sequences. Nodes supported with bootstrap values ≥80 are marked with black dots. Sequences from suspected XE cases are reported in red and indicated with an arrow.

**Figure 2:**
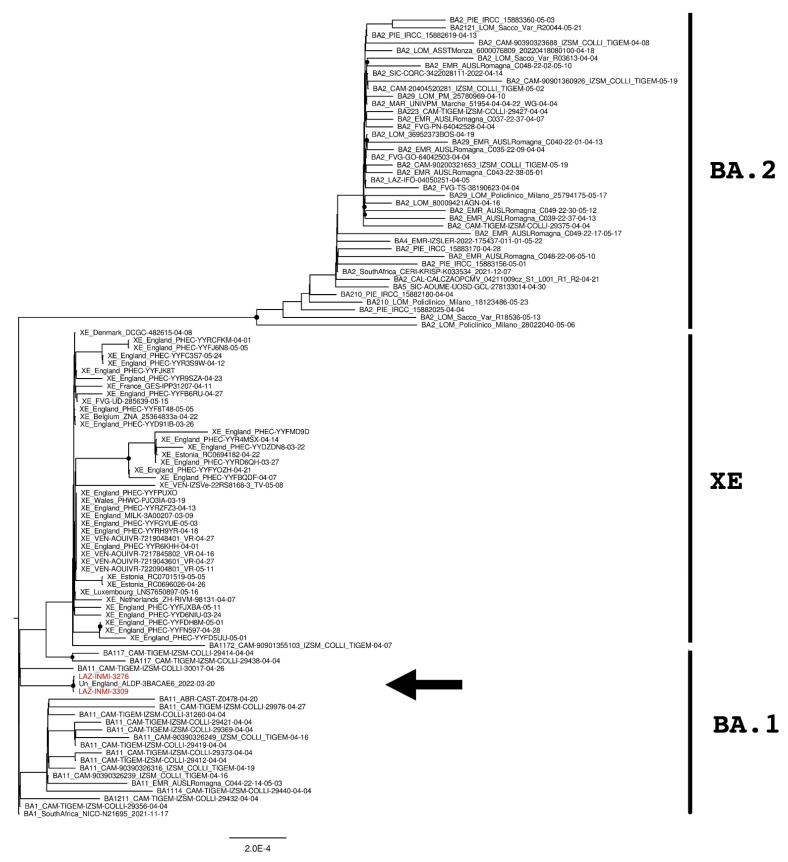
The phylogenetic tree is constructed by the maximum likelihood method and based on the XE recombination site: genomic elements from 266 to 11,500 nucleotides. Nodes supported with bootstrap values ≥80 are marked with black dots. Sequences from suspected XE cases are reported in red and indicated with an arrow.

**Figure 3:**
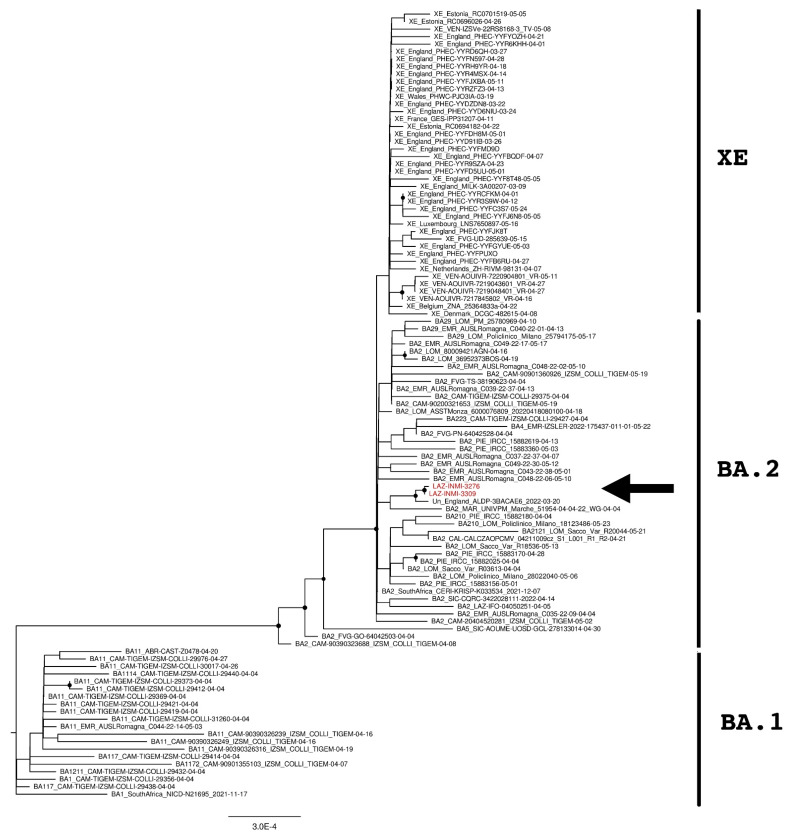
The phylogenetic tree is constructed by the maximum likelihood method and based on the XE recombination site: genomic element from 11,501 to 29,500. Untranslated regions are excluded from the analysis. Nodes supported with bootstrap values ≥80 are marked with black dots. Sequences from suspected XE cases are reported in red and indicated with an arrow.

Phylogenetic analyses were then performed to verify the genomic similarity with sequences found in Europe and Italy. Multiple phylogenetic trees were built by using the whole genome (Figure 1) and the upstream and downstream regions of the XE recombination site (Figure 2 and Figure 3) [19]. The trees were performed by using 139 sequences, including Italian XE, European XE, unclassified strains, BA.1, BA.2, BA.4, and BA.5 sequences.

The phylogenetic tree of the full-length sequences in Figure 1 revealed that INMI strains clustered together with an “unclassified” sequence from England (Accession ID: EPI_ISL_11446668) and that these three sequences were phylogenetically separated from the European and Italian XE groups with a significant bootstrap (>80% bootstrap replicates).

SARS-CoV-2 genomes were then divided into the upstream part and downstream part, corresponding to the genomic element from 266 to 11,537 and from 11,501 to 29,674 nucleotides, respectively. Two further phylogenetic analyses were then performed for each genomic part and reported in Figure 2 and Figure 3. As expected, XE sequences clustered close to BA.1 sequences in the upstream part of the recombination site (Figure 2) while clustered with BA.2 sequences in the downstream part (Figure 3). The same behavior was also observed for INMI strains but, as previously revealed by the whole-genome phylogenetic analysis, they resulted as phylogenetically separated from the European and Italian XE groups with a significant bootstrap (>80% bootstrap replicates) in both upstream and downstream regions. Moreover, both phylogenetic trees showed that INMI strains segregated in a well-separated clade with the same sequence from England, in the upstream and downstream parts of the XE recombination site.

Additionally, the distance analysis was calculated using the inter-groups and the intra-group genetic distances. The intra-group analysis revealed that the two INMI sequences were identical and that the diversity in the BA.1 and BA.2 groups was 4.97 × 10^−4^ and 4.01 × 10^−4^ nucleotide substitutions per site, respectively. The inter-groups analysis showed that INMI was more similar to the Italian and European XE groups than the BA.1 and BA.2 groups (Table 2), confirming that INMI sequences belonged to XE variants.

By the genetic distance analysis, the upstream region (658–11,500 nucleotides) resulted more similar to BA.1 than to BA.2 (4.23 × 10^−4^ and 1.45 × 10^−3^ nucleotide substitutions per site, respectively), while it was the opposite for the downstream region, spanning from 11,501 to 29,500 nucleotides with 1.68 × 10^−3^ and 3.86 × 10^−4^ nucleotide substitutions per site relative to BA.1 and BA.2, respectively.

Finally, the RDP4 analysis of INMI sequences showed a recombinant breakpoint between BA.1 and BA.2, approximately located at the nucleotide position 11,556 (99% confidence interval: 9460–13,866), that is in accordance with a previously observed XE recombinant breakpoint [19], and thus compatible with XE classification of INMI sequences by the Nextclade program (Figure S1).

## 4. Discussion

Despite the increasing use of viral genome sequencing for outbreak investigation of emerging or re-emerging pathogens in recent years, the size of genomic surveillance performed during the current SARS-CoV-2 pandemic is unprecedented. Genome sequencing has been used for studying the origin of the virus, identifying the emergence of genetic mutations into the viral genome, and monitoring the epidemiological trends of the novel emerging variants. The importance and utility of Whole-Genome Sequencing for tracking and understanding the SARS-CoV-2 pandemic are now abundantly clear, and several countries around the world have developed a massive genomic effort based on a high sequencing capacity for improving the routine global surveillance of potential variants. Accordingly, a national flash survey based on Whole-Genome Sequencing has been designed and implemented for monitoring the circulation of SARS-CoV-2 variants in Italy [34,35].

During Whole-Genome Sequencing analyses run in April and May 2022, according to Italian surveys, we identified two XE recombinant sequences, the first ones detected in Central Italy. This finding is of interest because, as well as for other RNA viruses, recombination represents an important contributor to SARS-CoV-2 evolution [36]. Many SARS-CoV-2 recombinant strains have been described in several countries, mostly those having denser genomic surveillance programs. Albeit recombination extremely likely occurs between SARS-CoV-2 lineages, recombinants can be difficult to detect whenever the sublineages have minimal differences and require Whole-Genome Sequencing efforts, given that, as for XE, sequencing of the spike gene only is too limited for detecting recombination. Additionally, only a few recombinant strains are likely detected since most of them are unlikely to be fitter than the currently dominant variant. The finding of two identical XE strains at different time points and from patients with no evident epidemiological link could indicate the persistence of this recombinant variant in our geographic area and the existence of a buried number of circulating XE strains to be still identified. The identity between INMI XE strains and the XE-like sequence found in England confirmed that our sequences were not an artifact and that the cluster of these XE-like sequences circulates in Europe.

The laboratory molecular methods applied for identifying SARS-CoV-2 lineages are often targeted to the Spike region only. This could lead to a misclassification of the variant, given that for XE identification, spike gene sequencing is not enough to detect the recombination event. Additionally, recombinant variants produced by uncanonical recombination events could be identified as “undetermined” lineage using the most popular online programs of SARS-CoV-2 lineages classification. Thus, without additional bioinformatic analyses, these cases may be lost. Finally, molecular tests are increasingly being replaced by rapid antigenic tests, which are the cheapest option in terms of time and cost, but also the worst option for identifying the lineage and monitoring the variants spread. All these criticisms require urgent and massive plans in order to perform more accurate genome surveillance by Whole-Genome Sequencing to monitor the emergence and spread of recombinant lineages [15]. Very little is still known about the recombinant variants, thus more research is needed to better understand their properties, such as infectivity, re-infectivity, immune escape, epidemiological and clinical data to develop appropriate control strategies. Currently, no recombinant lineage has shown the potential to grow fast enough to become dominant, and greater concern comes from the emerging BA.5 sublineages. However, among recombinants, XE has been shown to be fit enough to compete with BA.2; therefore, it follows the importance of monitoring its circulation as well as that of early identifying the eventual emergence of new recombinant variants.

## 5. Conclusions

Rapid public health response to the SARS-CoV-2 pandemic requires deep knowledge of how the virus is changing over time and how it is circulating over territory. Whole-Genome Sequencing has been used to trace in real time the SARS-CoV-2 evolution starting from the beginning of the pandemic in 2020. Millions of individual SARS-CoV-2 genomes have been sequenced and published in several international bioinformatics databases, such as GISAID and NCBI. As each new variant emerged, genomic surveillance has guided public health decisions, updated guidelines, and maximized activities for vaccine and drug development. Thus, the accurate identification of the SARS-CoV-2 variants is critical for the new emerging strain surveillance, and the Whole-Genome Sequencing by NGS plays a crucial tool in accurately detecting the emerging new variants. In this report, we describe the identification by Whole-Genome Sequencing of two SARS-CoV-2 strains that belonged to the rare clade XE. Importantly, although they contained genomic elements from BA.1 and BA.2 lineages, our SARS-CoV-2 XE sequences were phylogenetically distinct from the other European XE strains, although showing a similar recombinant breakpoint. By the analyses of the SARS-CoV-2 sequences published in international genetic sequence databases, we identified a similar strain found in England in March, thus remarking that this clade of XE-like strains is real and present in Europe. In conclusion, our study points out the importance of the implementation of the Whole-Genome Sequencing approach to monitoring the circulation of SARS-CoV-2 variants, indicating that the identification of XE-like variants often requires specific tools and advanced bioinformatic skills.

## Figures and Tables

**Table 1:** Demographic and clinical characteristics of the patients.

	Gender	Age (Years)	Vaccination	Symptoms Onset (Date)	Clinical Features	Admission (Date)	Discharge (Date)
Pt 1	Female	81	None	19 April 2022	Pneumonia	20 April 2022	4 May 2022
Pt 2	Male	59	Pfizer-BioNTech *	24 May 2022	Fever, myalgia, arthralgia, rash, asthenia, anorexia, cough, syncope	25 May 2022	1 June 2022

* 2 doses, the last on 21 October 2021.

**Table 2:** Inter-group genetic distances calculated over the entire genome.

	BA.1	BA.2	INMI	XE Eu	XE It
**BA.1**		*2.06 × 10^−4^* ^1^	*1.84 × 10^−4^*	*1.90 × 10^−4^*	*1.94 × 10^−4^*
**BA.2**	1.74 × 10^−3^		*1.36 × 10^−4^*	*1.28 × 10^−4^*	*1.33 × 10^−4^*
**INMI**	1.22 × 10^−3^	7.76 × 10^−4^		*9.68 × 10^−4^*	*1.03 × 10^−4^*
**XE Eu**	1.34 × 10^−3^	7.95 × 10^−4^	3.57 × 10^−4^		*3.68 × 10^−5^*
**XE It**	1.35 × 10^−3^	7.87 × 10^−4^	3.66 × 10^−4^	1.75 × 10^−4^	

^1^ distance standard deviations are reported in the upper part of the table, in italics.

## Data Availability

The sequences have been deposited in GISAID and GenBank with accession IDs EPI_ISL_12739848 and EPI_ISL_13300253.

## Supplementary Materials

The following supporting information can be downloaded at: https://www.mdpi.com/article/10.3390/diagnostics12092219/s1, Figure S1: RDP4 recombination plot.
