# Supplementary figures and images for: Molecular Characterization of Whole-Genome SARS-CoV-2 from the First Suspected Cases of the XE Variant in the Lazio Region, Italy
Source: Diagnostics (Basel). 2022 Sep 14;12(9):2219. doi: 10.3390/diagnostics12092219 (PMC9497488; doi:10.3390/diagnostics12092219)

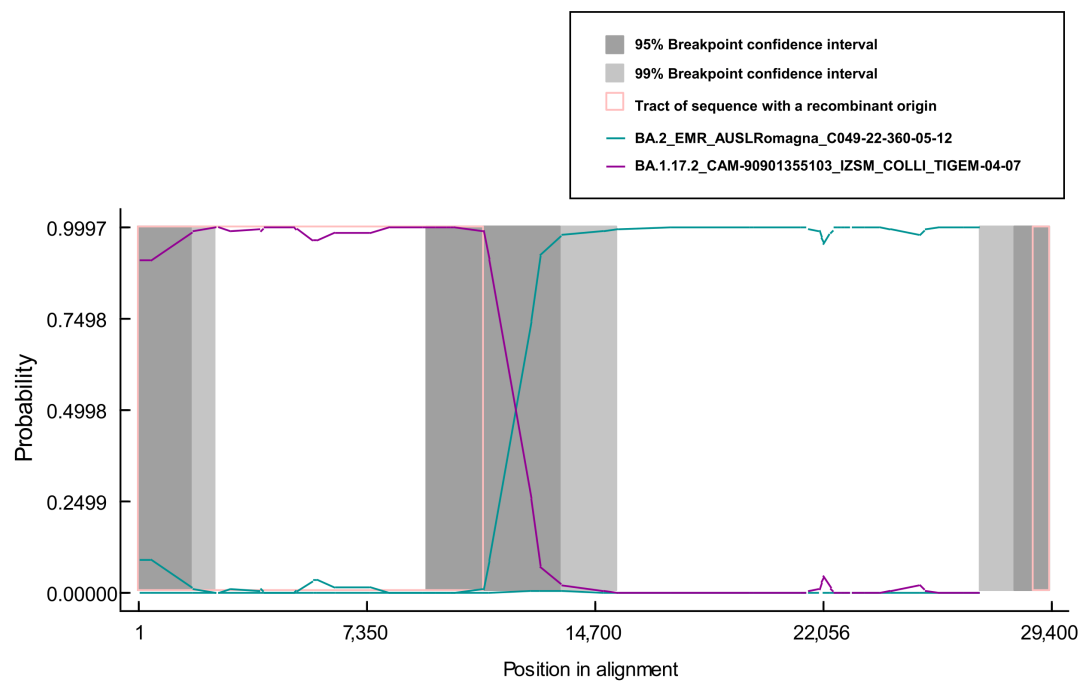

**Figure S1.** RDP4 recombination plot.

Supplement: Supplementary file 1 [file diagnostics-12-02219-s001.zip › diagnostics-1854152-supplementary.pdf]
